# Distinction Between *Aspergillus oryzae* and Aflatoxigenic *Aspergillus flavus* by Rapid PCR Method Based on the Comparative Sequence Analysis of the Aflatoxin Biosynthesis Gene Cluster

**DOI:** 10.3390/jof12010010

**Published:** 2025-12-23

**Authors:** Eunji Jeong, Yoo Jin Kwon, Jeong-Ah Seo

**Affiliations:** School of Systems Biomedical Science, Soongsil University, Seoul 06978, Republic of Korea; wjddmswl4789@naver.com (E.J.); kkyyjj8@naver.com (Y.J.K.)

**Keywords:** aflatoxin biosynthesis gene cluster, comparative genome analysis, deletion patterns, newly designed primer sets, PCR method to distinguish between *A. oryzae* and *A. flavus*

## Abstract

*Aspergillus oryzae* and *Aspergillus flavus* are closely related species within the *Aspergillus* section *Flavi*, sharing approximately 99.5% genomic similarity. Despite this similarity, they differ markedly in their ability to produce aflatoxin, a carcinogenic mycotoxin synthesized by the aflatoxin biosynthesis gene cluster (ABGC). Species and strains included within section *Flavi* display diverse deletion patterns in the ABGC at the sequence level. In this study, we performed an in-depth comparative analysis of the ABGC of 30 strains belonging to section *Flavi*, including isolates obtained from *nuruk*. The analysis revealed that *A. oryzae* exhibits distinct large-scale or locus-specific deletions in the ABGC compared to other related species. Based on these unique deletion patterns, we designed four primer sets to distinguish *A. oryzae* from *A. flavus* by comparing the sizes of PCR amplicons. Application of these primer sets to *nuruk*-derived isolates enabled successful species differentiation with 92% accuracy. To further validate this method, in silico PCR analysis was conducted using publicly available genomes of *A. oryzae* (116) and *A. flavus* (482), confirming that the developed biomarkers could consistently distinguish between the two close species. The primer sets are expected to serve as a rapid, accurate, and practical method for distinguishing *A. oryzae* from *A. flavus*.

## 1. Introduction

*Aspergillus* section *Flavi* comprises 34 species that are morphologically and phylogenetically related and are classically divided into two groups based on their effects on food and human health [1,2,3,4]. The first group includes non-aflatoxigenic species such as *Aspergillus oryzae* and *Aspergillus sojae*, which have been domesticated and traditionally used in East Asia for the production of fermented foods and beverages [5,6,7]. In particular, *A. oryzae* is industrially important for its production of catalytic enzymes such as amylase and has been designated as “generally recognized as safe (GRAS)” by the U.S. Food and Drug Administration (FDA) and the World Health Organization (WHO), underscoring its broad industrial utility [8,9,10]. In contrast, the second group consists of aflatoxigenic species such as *Aspergillus flavus* and *Aspergillus parasiticus*, which produce carcinogenic aflatoxins that contaminate agricultural products and food products, posing significant public health concerns [11,12,13,14]. In addition to aflatoxin-related toxicity, *A. flavus* is recognized as one of the world’s most problematic fungal pathogens, causing invasive aspergillosis in humans and opportunistic infections in major economic crops both pre- and post-harvest [15,16,17].

Precise identification of *Aspergillus* section *Flavi* at the species level is critical because it includes both non-aflatoxigenic GRAS-certified species used in the food industry and aflatoxin-producing species threatening food safety [3,18,19,20,21,22]. Molecular biological analyses, including marker sequence comparison and genomic polymorphism assessments, have been used for this purpose [3,23]. However, these approaches generally distinguish between *A. flavus*/*A. oryzae* group and *A. parasiticus*/*A. sojae* group, but are still inadequate for distinguishing *A. oryzae* from *A. flavus* [24,25,26]. *A. oryzae* and *A. flavus*, two representative species of *Aspergillus* section *Flavi* with opposite impacts on the food industry, share nearly identical morphological traits and differ by only 0.5% (approximately 200 kb) of their genomic sequences [27,28,29]. Distinguishing the two species is difficult using only mycological or traditional molecular approaches, and therefore requires an integrated analysis combining morphological comparison, molecular genetic identification, phylogenetic analysis, and aflatoxin production assessment [4,30,31].

Although *A. oryzae* and *A. flavus* share a high degree of genetic similarity, their aflatoxin-producing abilities differ fundamentally. For this reason, numerous studies have been conducted to elucidate the genetic basis for these differences in toxin production through genetic analysis of the aflatoxin biosynthesis gene cluster (ABGC) [27,32,33]. The ABGC comprises 34 *afl* genes involved in the aflatoxin biosynthetic pathway or in adjacent sugar utilization, organized within an approximately 75 kb of DNA region at the terminus of chromosome 3 in *Aspergillus* section *Flavi* [34,35]. Comparative genomic analyses of the ABGC in *A. oryzae* and *A. flavus* have revealed strain-specific deletions at both gene and nucleotide levels across strains [25,36,37]. In particular, *A. flavus* exhibits deletions within the *aflU* (*cypA*)-*aflF* (*norB*) genes that are 0.8 to 1.5 kb shorter than the corresponding region in G-type aflatoxin-producing *A. parasiticus* [38,39,40]. Likewise, *A. oryzae* exhibits unidirectional large deletions with various breakpoints across strains, resulting in partial retention of aflatoxin-related genes yet loss of toxin production capacity [41,42,43]. Phylogenetic analyses of ABGC sequences further suggest that *A. oryzae* is phylogenetically closer to non-aflatoxigenic *A. flavus* than to aflatoxigenic *A. flavus*, implying that the most recent ancestor of *A. oryzae* diverged from a non-aflatoxigenic *A. flavus* [44,45]. Nonetheless, although these studies have attempted to distinguish between *A. oryzae* and *A. flavus* by large-scale polymerase chain reaction (PCR) and sequencing of a few selected genes, a suitable set of genes to distinguish both species has not yet been found [30,41,46]. Therefore, the development of precise markers and concise methods is essential for differentiating these closely related species for reliable industrial application of beneficial strains and food quality assurance [47,48].

In our previous studies, we have performed comparative genomic analyses of 30 representative species belonging to *Aspergillus* section *Flavi*, including *A. oryzae* and *A. flavus* strains isolated from *nuruk*, a traditional Korean fermentation starter [49,50,51]. Through these analyses based on the sequence level, we found the strain-specific deletion patterns within the ABGC, providing the basis for the present study. Based on the results, we aimed to design the primer sets to distinguish *A*. *oryzae* from *A*. *flavus* by utilizing the differences in specific gene sequences within the ABGC. To evaluate the diagnostic potential of these primers, we performed PCR analysis on 108 *A*. *oryzae* strains isolated from *nuruk*. In addition, we downloaded 116 genomes of *A. oryzae* and 482 genomes of *A*. *flavus* registered in GenBank and AflaPan [52] and performed in silico PCR analysis using the designed primers, and confirmed that these primers could distinguish between the two highly similar species. This study was conducted to contribute to the development of a rapid and highly accurate identification method for distinguishing between *A*. *flavus* strains that produce aflatoxins and threaten food safety from *A*. *oryzae*, which is recognized as GRAS, by utilizing the results of more sophisticated and in-depth comparative genomic analysis.

## 2. Materials and Methods

### 2.1. Comparison of ABGC Genes of 30 Strains of Aspergillus Section Flavi

The whole genome sequences of 30 strains of *Aspergillus* section *Flavi*, previously analyzed in a comparative genome study [49], were retrieved from GenBank, and in this study, a comparative analysis of the ABGC sequences was performed. The ABGC sequences of 30 strains of *Aspergillus* section *Flavi* were obtained via BLASTn (NCBI, https://blast.ncbi.nlm.nih.gov/Blast.cgi, accessed on 1 November 2024) [53], using the annotation information of ABGC of *A. flavus* NRRL3357 as a reference (GCA_014117465.1). The 35 ABGC genes analyzed in this study consist of 5 sugar utilization-related genes (*aflYa* to *aflYe*) [54,55], 29 genes associated with aflatoxin biosynthesis (*aflA* to *aflY*, *aflCa*, *aflLa*, *aflMa*, and *aflNa*) [56], and AFLA_13945 gene (EDD51179.1), a hypothetical protein-coding gene located in the 3′ end of ABGC. The ABGC sequences of all strains were multiply aligned using the DIALIGN program v2.2.2 [57], and information on frameshifts was obtained using MACSE v1 [58]. The average nucleotide identity (ANI) values among the ABGC sequences of 30 strains of *Aspergillus* section *Flavi* were calculated using OrthoANI v0.6.0 [59]. A phylogenetic tree based on ANI values was constructed using the hclust function of the R program v4.3.2.

### 2.2. PCR Analysis of A. oryzae and A. flavus Strains Using Newly Designed Four Sets of Primers Targeting ABGC

Four primer sets were designed to target four regions within the ABGC, namely YE1–YE2, CT1–CT2, UF1–UF2, and E1–E2. Each primer targeted corresponding positions: positions 304–621 of *aflYe* gene (start region), positions 70,774–72,500 of the *aflC* and *aflT* genes (CT region), positions 74,498–76,669 of the *aflU* and *aflT* genes (UF region), and positions 77,621–77,942 of the AFLA_13945 (end region), the terminal ABGC gene (Table 1). The annealing temperatures for each primer set were 64.8, 65.2, 64.9, and 64.2 °C, respectively. Each primer was designed to target conserved regions of *A. oryzae* and *A. flavus*, but with 1–4 sequence variations per species (Table S1).

One hundred seventeen strains that formed green spores were isolated from *nuruk*, and identified by molecular methods using internal transcribed spacer (ITS) region, and confirmed with high-performance liquid chromatography (HPLC) analysis for aflatoxin production [51,60,61]. Aflatoxin production of *nuruk* strains was measured by culturing them on rice medium at 25 °C for 21 days. Aflatoxin was extracted from the rice medium using an immunoaffinity column (*AflaTest*^®^WB; VICAM, A Waters Business, San Marcos, CA, USA). The final concentration of aflatoxin was measured by HPLC analysis using a 1200 Infinity Series (Agilent Technologies, Santa Clara, CA, USA) on a ZORBAX Eclipse Plus C_18_ column (4.6 × 150 mm, 3.5 µm). HPLC analysis through a C_18_ column was performed at a flow rate of 1.0 mL/min using an isocratic mobile phase composed of water, methanol, and acetonitrile in the ratio of 50:40:10 (*v*/*v*/*v*). Aflatoxins were detected using a fluorescent detector with an excitation wavelength of 360 nm and emission wavelength of 440 nm.

We performed PCR analysis using four newly designed primer sets on 108 strains of *A. oryzae* and 9 strains of *A. flavus* isolated from *nuruk* and 8 standard strains of *Aspergillus* section *Flavi*. PCR reactions were carried out at a final volume of 20 µl, containing 20 to 50 ng of genomic DNA of each strain and 20 pmol of each primer. The reactions were conducted under the following conditions: 94 °C for 4 min, followed by 30 cycles of 94 °C for 1 min, 65 °C for 1 min, and 72 °C for 1 min, and a final extension at 72 °C for 10 min. The amplified PCR amplicons were confirmed by electrophoresis using a 1.0% agarose gel.

### 2.3. Comparison of laeA Gene and Translated LaeA Sequences

Draft annotation for *laeA* gene was conducted on 116 genomes of *A. oryzae* and 257 genomes of *A. flavus* obtained from the NCBI GenBank database and 225 genomes of *A. flavus* from AflaPan [52] using the Funannotate pipeline v1.8.15 [62]. The nucleotide sequence of *laeA* gene and the amino acid sequence of LaeA for each strain were subjected to BLAST to detect sequence variations. The *laeA* gene sequences showing sequence variations were subjected to multiple alignment using MAFFT v7.407 [63,64], and the positions of sequence variations and mutation cases were examined.

### 2.4. In Silico PCR Analysis Using ABGC-Targeting Primer Sets

To perform in silico PCR (iPCR) analysis using four primer sets, we acquired whole genome sequences of 116 strains of *A. oryzae* and 257 strains of *A. flavus* registered in GenBank. In addition, we independently acquired the newly sequenced genome sequences of 225 strains of *A. flavus* from the AflaPan [52]. And then, we performed iPCR analysis using Fasta v36.3 [65] to determine the target positions and expected PCR amplicon sizes for a total of 116 genomes of *A. oryzae* and 482 genomes of *A. flavus*. To confirm the sequence variations of the ABGC-targeting primer sets, we extracted the aligned sequences from 116 genome of *A. oryzae* and 482 genomes of *A. flavus* with newly designed primer sets, and conducted multiple alignment using MAFFT v7.407 [63,64]. Finally, the alignment of the four primer sets with the genomes of *A. oryzae* and *A. flavus* was conducted using BLAST [66] to validate the expected iPCR amplicon size and sequence variations within the primer sets.

## 3. Results

### 3.1. Comparison of ABGC Among 30 Strains of Aspergillus Section Flavi

We acquired the ABGC sequences from the genomic data of 30 strains of *Aspergillus* section *Flavi* and examined the gene deletions and pseudogenization across 35 ABGC genes through additional annotation (Table S2). The number of ABGC genes varied among *A. oryzae* and *A. flavus* strains, whereas *A. sojae* and *A. parasiticus* possessed the whole set of 35 genes. Nine strains of *A. oryzae* possessed all 35 ABGC genes, including 6 to 11 pseudogenes. In contrast, ten *A. flavus* strains and two *A. oryzae* strains each harbored 33 to 35 ABGC genes, with the *aflT*, *aflU*, and *aflF* genes exhibiting diverse statuses, such as intact, deleted, or pseudogene. The remaining four strains of *A. oryzae* and *A. flavus* exhibited unidirectional large deletions starting at the 3′ end of ABGC. Specifically, *A. oryzae* KBP3 and BCC7052 retained 21 genes due to deletions spanning *aflE* to AFLA_13945, *A. oryzae* RIB326 retained 10 genes due to deletions from *aflQ* to AFLA_13945, and *A. flavus* 26-3 contained only 4 genes resulting from deletions extending from *aflYa* to AFLA_13945.

**Figure 1:**
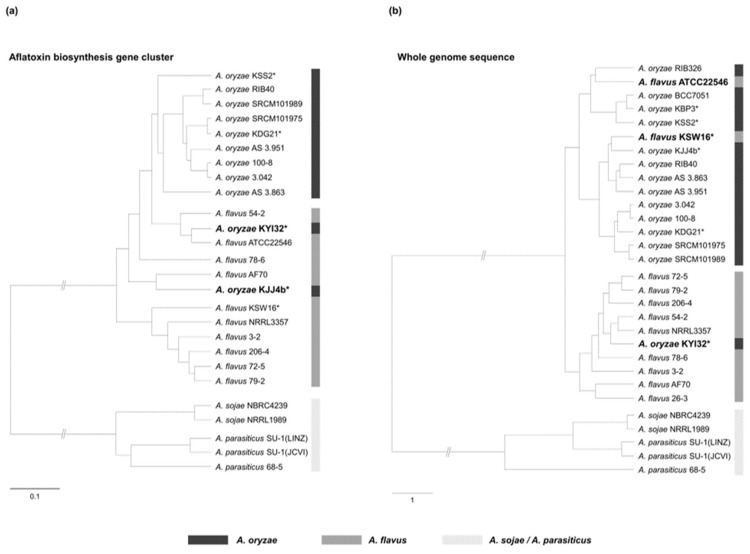
Phylogenetic tree of 30 strains of *Aspergillus* section *Flavi*. Maximum likelihood phylogenetic analysis was conducted using (**a**) the full sequences of ABGC and (**b**) the whole genome sequences of 30 strains of *Aspergillus* section *Flavi*. The whole genome sequences of 22 strains were obtained from the GenBank, while the others were derived from our previous study [49]. Four strains (*A. oryzae* KBP3, RIB326, BCC7051, and *A. flavus* 26-3) possessing large deletions in the ABGC are excluded from the ABGC-based phylogenetic tree. An asterisk (*) indicates strains isolated from *nuruk*, and strains belonging to another clade are shown in bold.

A phylogenetic analysis was conducted using the ABGC sequences of 26 strains of *Aspergillus* section *Flavi*, excluding four strains (*A. oryzae* KBP3, RIB326, BCC7051, and *A. flavus* 26-3) that had unidirectional deletions exceeding one-third of the ABGC (Figure 1a). The ABGC-based phylogenetic tree was divided into three primary clades corresponding to *A. oryzae*, *A. flavus*, and *A. sojae*/*A. parasiticus*. The *A. oryzae* clade contained nine *A. oryzae* strains, whereas the *A. flavus* clade comprised ten *A. flavus* strains and two *A. oryzae* strains (Figure 1a). As indicated in bold in Figure 1a, both *A. oryzae* KYI32 and KJJ4b, which originated from *nuruk*, clustered within the *A. flavus* clade. Interestingly, while *A. oryzae* KYI32 consistently belonged to the *A. flavus* clade in both the ABGC-based and whole-genome-based phylogenetic trees, *A. oryzae* KJJ4b belonged to the *A. oryzae* clade in whole-genome phylogeny (Figure 1b).

### 3.2. Five Deletion Types of the ABGC Across 30 Strains of Aspergillus Section Flavi

**Figure 2:**
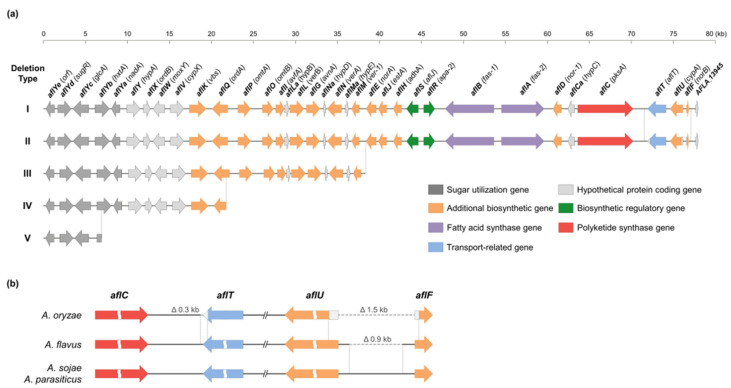
Structural comparison of 35 genes in ABGCs of *Aspergillus* section *Flavi*. (**a**) The structural differences of 35 ABGC genes, spanning approximately 78 kb on 3′ end of chromosome 3, are schematically depicted. The 30 strains of *Aspergillus* section *Flavi* were categorized into five deletion types based on structural differences of 35 genes in ABGC. Deletion type I includes 2 strains of *A. oryzae* (KYI32 and KJJ4b) and 8 strains of *A. flavus* (54-2, ATCC22546, 78-6, NRRL3357, 3-2, 106-4, 72-5, and 79-2); type II includes 9 strains of *A. oryzae* (KSS2, RIB40, SRCM101975, SRCM101989, KDG21, AS3.951, 100-8, 3.042, and AS3.863) and 2 strains of *A. flavus* (AF70 and KSW16); type III includes 2 strains *A. oryzae* KBP3 and BCC7051; type IV includes *A. oryzae* RIB326; type V includes *A. flavus* 26-3. The arrows indicate the direction of gene transcription. The color of arrow indicates the category of enzyme or product associated with aflatoxin biosynthesis, as encoded by the corresponding gene. The new and old nomenclatures for each gene in ABGC are presented in bold and in parentheses above each gene, respectively [67]. (**b**) Species-specific small deletions in *aflT*, *aflU* (*cypA*), and *aflF* (*norB*) genes were depicted. Deletion type II *A. oryzae*, which possessed all 35 ABGC genes, exhibited approximately 0.3 kb deletions from the 3′-end of the *aflT* gene to the intergenic region to the *aflC* gene in comparison to *A. flavus*, *A. sojae* and *A. parasiticus*. And *A. oryzae* exhibited a deletion in the intergenic region including the 5′-end of the *aflU* and *aflF* genes, resulting in the *aflU*-*aflF* region being about 1.5 kb shorter than that of *A. sojae* and *A. parasiticus*, and 0.6 kb shorter than that of *A. flavus*. In the stance of *A. flavus*, a deletion was present in the intergenic region of *aflU* and *aflF,* resulting in the *aflU*-*aflF* region being 0.9 kb shorter than that of *A. sojae and A. parasiticus*.

Based on the distribution of 35 ABGC genes in 30 strains of *Aspergillus* section *Flavi* (Table S2), ABGC deletion patterns were classified into five types (Figure 2a). Deletion type I contains 33 to 35 ABGC genes, and two strains of *A. oryzae*, 10 strains of *A. flavus*, 2 strains of *A. sojae*, and 3 strains of *A. parasiticus* belong to this type (Table S2). In the case of deletion type II, the *aflT*, *aflU*, and *aflF* genes located at the 3′ end of ABGC were pseudogenes, and 9 strains of *A. oryzae* belong to this type. Deletion types III, IV, and V are minor types in which more than half of the ABGC is deleted; deletion type III has 21 ABGC genes, type IV has 10 ABGC genes, and type V has 4 ABGC genes.

*Aspergillus* section *Flavi* strains classified as deletion type I and II exhibited two species-specific deletions involving three adjacent genes—*aflT*, *aflU*, and *aflF*—at the 3′ end of the ABGC (Figure 2b). The first species-specific deletion occurred within the *aflT* gene and the intergenic region between *aflC* (*pksA*) and *aflT*. This deletion spanned approximately 0.3 kb, encompassing about 250 bp from the 3′ end of *aflT* and roughly 60 bp from the *aflC*-*aflT* intergenic region, and was observed exclusively in *A. oryzae*. The second species-specific deletion was located in the *aflU* (*cypA*) gene, *aflF* (*norB*) gene, and their intergenic region. In *A. sojae* and *A. parasiticus*, this intergenic region measured approximately 1.4 kb, whereas *A. flavus* exhibited a 0.9 kb deletion, leaving only 0.5 kb. In *A. oryzae*, a more widespread deletion occurred, including the *aflU*-*aflF* intergenic region and the 5′ end of two genes, leading to a more significant deletion of approximately 1.5 kb compared to *A. sojae* and *A. parasiticus* and 0.6 kb relative to *A. flavus* (Figure 2b).

### 3.3. PCR-Based Differentiation of A. oryzae from A. flavus Using ABGC-Targeting Primer Set

**Figure 3:**
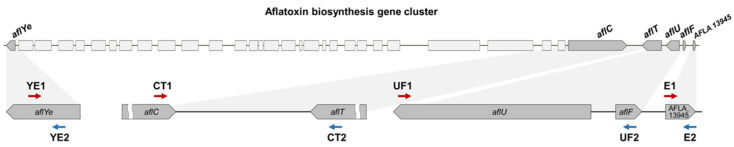
Target location of the four newly designed primer sets on ABGC. The target location of the primer sets designed to differentiate *A. oryzae* and *A. flavus* based on the differences in gene sequences within ABGC of two species was depicted. The four primer sets comprise as follow; YE1 and YE2 pair and E1 and E2 pair which target the *aflYe* gene (start) and AFLA_13945 gene (end), respectively, and CT1 and CT2, UF1 and UF2, which target the *aflC*–*aflT* (CT) region and the *aflU*–*aflF* (UF) region, where differences in gene sequences between *A. oryzae* and *A. flavus* are present, respectively. The nucleotide positions of the target locations for the primer sets are shown in Table 1, and accordingly, the locations of these primer sets were illustrated. The red and blue arrows indicate forward and reverse primers, respectively. The gray arrows indicate the transcriptional direction of ABGC genes, and black lines represent intergenic regions.

Based on the species-specific deletion types identified within ABGC of *Aspergillus* section *Flavi*, we designed four primer sets to distinguish between *A. oryzae* and *A. flavus* through a simple PCR assay comparing amplicon sizes (Table 1). The newly designed primer sets—YE1–YE2, CT1–CT2, UF1–UF2, and E1–E2—targeted four distinct regions across the ABGC (Figure 3). The YE1-YE2 set targeted the *aflYe* gene (start region), the first gene of ABGC, allowing detection of the presence or absence of ABGC in the strain. The CT1–CT2 set targeted the *aflC* and *aflT* genes (CT region) and was designed to detect the 0.3 kb deletion unique to *A. oryzae*. The UF1–UF2 set targeted the *aflU* and *aflF* genes (UF region) to identify species-specific differences in deletion size of 0.6 kb between *A. oryzae* and *A. flavus*. The E1–E2 set targeted the AFLA_13945 gene (end region), the last gene of ABGC, to determine the presence or absence of large deletions within it.

**Figure 4:**
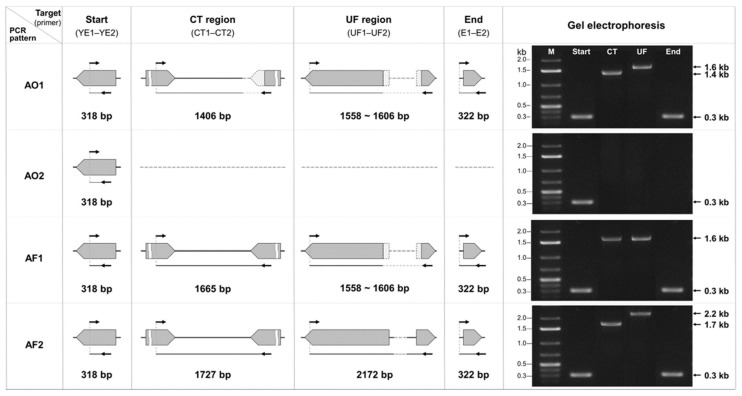
PCR pattern of *A. oryzae* and *A. flavus* using the newly designed primer sets. The four PCR patterns and corresponding amplicon sizes for four regions in ABGC, as determined by PCR analysis using ABGC-targeting primer sets, are illustrated. The gray arrows and black lines each represent the gene and intergenic region, respectively. Light gray boxes and dashed lines represent deleted genes and deleted intergenic regions, respectively. In the AO1 pattern, all four regions are amplified, with the amplicon sizes for the CT and UF regions being around 1.4 and 1.6 kb, respectively. The AO2 pattern refers to the amplification of just the *aflYe* gene (start). For the AF1 pattern, all four regions are amplified, with the amplicon sizes for the CT and UF regions being around 1.7 and 1.6 kb, respectively. In the AF2 pattern, all four regions are amplified, with the amplicon sizes for the CT and UF regions being around 1.7 and 1.6 kb, respectively. The amplicon sizes for four PCR patterns were verified using electrophoresis on 1% agarose gel. The representative strains examined to verify the PCR amplicon sizes for each pattern were *A. oryzae* KSS2, KBP3, *A. flavus* KSW16, and ATCC22546. The marker (M) utilized for electrophoresis was GeneRuler 1 kb plus DNA ladder.

PCR assay using these four primer sets revealed two distinct amplification patterns for *A. oryzae* and *A. flavus* based on the CT and UF regions: AO1 and AO2 patterns for *A. oryzae*, and AF1 and AF2 patterns for *A. flavus* (Figure 4). Across all patterns, the amplicon sizes of the start and end regions were 318 bp and 322 bp, respectively, except for AO2, in which the end region was not amplified. In the AO1 pattern, the CT and UF regions produced amplicons of 1406 bp and ~1600 bp, respectively, corresponding to *A. oryzae* strains carrying a 0.3 kb deletion in CT region and a 0.6 kb deletion in the UF region. The AO2 pattern displayed amplification only in the start region, with the other three regions unamplified, consistent with *A. oryzae* strains exhibiting large deletion within ABGC (deletion type III to V in Figure 2a). In the case of *A. flavus*, the AF1 pattern produced CT and UF amplicons of 1665 bp and ~1600 bp, respectively, reflecting a 0.6 kb deletion in the UF region similar to that observed in *A. oryzae*. The AF2 pattern, on the other hand, generated CT and UF amplicons of 1727 bp and 2172 bp, respectively, corresponding to *A. flavus* strains classified as deletion type I in Figure 2a. PCR analysis using the four primer sets on representative strains—*A. oryzae* KSS2, KBP3, *A. flavus* KSW16, and ATCC22546—confirmed that these strains exhibited the AO1, AO2, AF1, and AF2 patterns, respectively (Figure 4).

### 3.4. PCR Analysis Using Four Newly Designed Primer Sets to Distinguish A. oryzae from A. flavus

We performed PCR analysis using the four primer sets on 125 strains, comprising 8 reference strains of *Aspergillus* section *Flavi*, 108 *A. oryzae* strains, and 9 *A. flavus* strains isolated from *nuruk*, alongside HPLC analysis to evaluate aflatoxin production for each strain (Table 2 and Table S3). PCR and HPLC analyses of the 8 reference strains revealed that *A. oryzae* RIB40 and RIB128, both non-aflatoxin-producers, exhibited the AO1 pattern, whereas *A. flavus* ATCC22546 and *A. flavus* NRRL3357, which produced about 300 ppb of aflatoxin, showed the AF2 pattern (Table S3 and Figure S1). Among the reference strains, PCR results of the two non-aflatoxin-producing *A. sojae* strains and the two aflatoxin-producing *A. parasiticus* strains both produced amplicons of the same size, 1.7 kb and 3.1 kb, in the CT and UF regions, respectively.

PCR analysis of 108 *nuruk* isolates of *A. oryzae* revealed 35 strains (32%) as AO1, 64 strains (59%) as AO2, 8 strains (7%) as AF1, and one strain (1%) as AF2 (Table 2). Eight strains isolated from *nuruk* (CN4-3, JJ4-7, JJ4-10, JJ4R-B, KJJ4b, SW1-8, SW1-11Y, and YI2-1) produced amplicons of approximately 1.7 kb and 1.6 kb in the CT and UF region, respectively, showing the AF1 pattern. *A. oryzae* KYI32 made amplicons of about 1.7 kb and 2.1 kb in the CT and UF regions, respectively, showing an AF2 pattern. All *A. flavus* strains isolated from *nuruk* were aflatoxigenic, ranging from 22.8 to 279.7 ppb, and all showed the AF2 pattern, except for *A. flavus* KSW16 (AF1 pattern) (Table S3).

**Figure 5:**
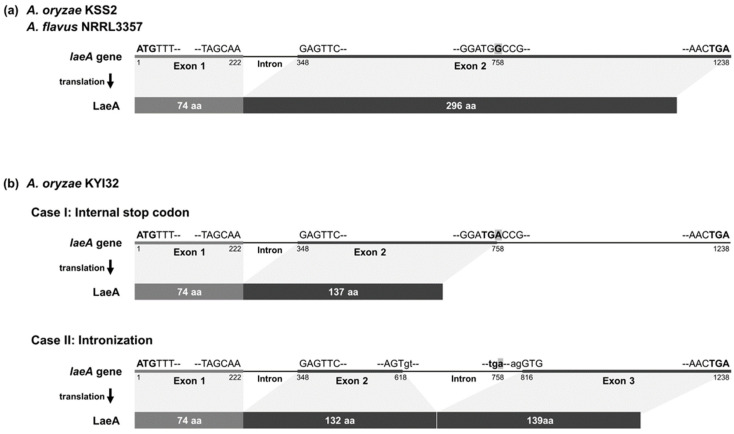
*laeA* gene and expected LaeA structure of *A. oryzae* KYI32. The substitution mutation in the *laeA* gene of *A. oryzae* KYI32 and the expected structure of LaeA, a global regulator of secondary metabolism of *Aspergillus* spp. [68], as predicted by BLAST, are schematically illustrated. In the *laeA* gene sequence, gray solid lines represent exons, whereas black lines represent introns. The location of mutation in sequence of *laeA* gene is highlighted in gray, and the start and stop codons are shown in bold. The expected sizes of LaeA are depicted in a gray box. (**a**) Compared to *A. oryzae* KSS2 and *A. flavus* NRRL3357, which have a normal *laeA* gene, (**b**) *A. oryzae* KYI32 had a substitution of guanine (G) with adenine (A) at 758th nucleotide position of *laeA* gene. BLAST analysis using *laeA* gene sequences of *A. oryzae* KYI32 indicated two anomalies in LaeA expression: an internal stop codon at 758th nucleotide position in the *laeA* gene, leading to the expression of truncated LaeA, or an internal intronization at 618th to 816th nucleotide position of *laeA* gene.

To further investigate atypical *A. oryzae* strains showing AF patterns, we compared the *laeA* gene sequences, a global regulator of secondary metabolism of *Aspergillus* spp. [68], in *A. oryzae* KJJ4b (AF1 pattern) and KYI32 (AF2 pattern) (Table 2). Compared with *A. oryzae* KSS2 and *A. flavus* NRRL3357, which possesses an intact *laeA* gene of 1238 bp, *A. oryzae* KYI32 has a single-nucleotide transition mutation at the 758th nucleotide position, where guanine (G) was substituted with adenine (A) (Figure 5). Based on *laeA* gene sequences of *A. oryzae* KSS2 and *A. flavus* NRRL3357, the predicted amino acid sequence of LaeA comprised 370 aa with no mutations (Figure 5a). In contrast, *A. oryzae* KYI32 exhibited two atypical translation cases of LaeA due to a G to A substitution (Figure 5b). Case I is a predicted truncated LaeA (210 aa) resulting from a premature stop codon at the 758th nucleotide, while case II is a predicted shortened LaeA (345 aa) caused by intronization between the 618th and 816th nucleotides of the *laeA* gene. However, the *laeA* gene of *A. oryzae* KJJ4b did not have any sequence mutations.

### 3.5. iPCR Analysis Using Four Newly Designed Primer Sets to Distinguish A. oryzae from A. flavus

To verify that the two species can be distinguished based on PCR, we performed iPCR analysis using 116 genomes of *A. oryzae* and 482 genomes of *A. flavus* available from GenBank and AflaPan [52] (Table 3, Tables S4 and S5). Most of the 116 *A. oryzae* genomes showed either the AO1 (43 strains, 37%) or the AO2 patterns (71 strains, 61%). The only exceptions were *A. oryzae* KJJ4b (AF1) and *A. oryzae* KYI32 (AF2) (Table 3). Subsequent iPCR analysis of 482 *A. flavus* genomes, including 257 from GenBank and 225 from AflaPan, revealed that 170 genomes (35%) showed the AF1 pattern and 146 genomes (30%) did the AF2 pattern. However, 24 genomes (5%) exhibited the AO1 pattern, and 121 genomes (25%) did the AO2 pattern.

All 225 genomes of *A. flavus* in AflaPan were tested for aflatoxin production status, but only 98 of the 257 genomes in GenBank were confirmed (Table S5). Consequently, we compared the iPCR results of 323 genomes (161 aflatoxigenic strains and 162 non-aflatoxigenic strains) with known aflatoxin production status (Table 3). The majority of aflatoxigenic *A. flavus* (95%) exhibited the AF1 or AF2 pattern, with only seven outliers. In the case of non-aflatoxigenic *A. flavus*, 76 genomes (47%) and 57 (35%) showed the AO2 and AF1 pattern, respectively.

## 4. Discussion

*Aspergillus* section *Flavi* includes both useful species, which are used in the food industry owing to their high enzyme productivity and fermentation efficiency, and harmful species, which synthesize carcinogenic mycotoxins that contaminate food products and pose serious health risks [70,71]. Owing to their industrial importance, species-level molecular identification within *Aspergillus* section *Flavi* remains one of the most extensively studied topics in fungal taxonomy [13,48,72,73]. However, *A. oryzae* and *A. flavus*, two closely related but functionally distinct species, are difficult to accurately distinguish based on morphology or common molecular markers such as ITS and β-tubulin sequences [4,27]. Since aflatoxin production represents the primary distinguishing trait between these two species, identification often relies on a comprehensive evaluation that incorporates aflatoxin production with morphological and molecular biological analyses [18,36,40]. However, such comprehensive analyses are time-consuming and complicated by the existence of non-aflatoxigenic *A. flavus* strains [30,48,74]. Therefore, a rapid and accurate molecular method is essential for detecting *A. flavus*, which continues to threaten global food safety and human health.

In our previous study [49], we found that *Aspergillus* section *Flavi* strains isolated from *nuruk*, a traditional Korean fermentation starter, possessed diverse ABGC deletion types, including truncated clusters, pseudogenes, and strain-specific deletions of ABGC genes. The genes involved in aflatoxin biosynthesis, which is the most prominent distinction between *A. oryzae* and *A. flavus,* are located as a cluster in the sub-telomeric region of chromosome 3, a region known for high frequencies of genetic recombination and structural rearrangements [38,75]. Several studies have reported that *Aspergillus* section *Flavi*, particularly *A. oryzae* and *A. flavus*, harbor species-specific structural variations in the ABGC, including large-scale gene deletions and small sequence-level deletions [33,43,72,76]. In this study, we analyzed ABGC deletion patterns at the sequence level in *A. oryzae* and related species within *Aspergillus* section *Flavi*, including *nuruk*-derived strains, to develop a reliable differentiation method to differentiate *A*. *oryzae* from *A*. *flavus*. Of the 14 *A. oryzae* strains analyzed, three exhibited unidirectional deletions starting from the 3′ end of the ABGC (Figure 2a and Table S2), consistent with the previously reported Group II deletion in *A. oryzae* [45,77]. *A. oryzae* KBP3 is a deletion type III, with the ABGC deleted up to the *aflM* gene, rendering it non-aflatoxigenic, similar to *A. oryzae* BCC7051 [45]. Most *A. oryzae* strains (9 out of 14) were deletion type II, characterized by a 0.3 kb deletion at the 3′ end of the *aflT* gene and a 1.5 kb deletion in the intergenic region encompassing the 5′ ends of the *aflU* and *aflF* genes (Figure 2b). These sequence-level deletions in the *aflT*, *aflU*, and *aflF* genes were confirmed as species-specific deletions of *A. oryzae* compared to the other three species, *A. flavus*, *A. sojae*, and *A. parasiticus* (Figure 1a) [38,41]. Unlike typical *A. oryzae* strains, *A. oryzae* KYI32 was phylogenetically grouped with *A. flavus* based on both ABGC sequences and the whole genome (Figure 1 and Table S2) [78,79]. Although *A. oryzae* KYI32 does not produce aflatoxins, as confirmed by HPLC analysis, its ABGC organization closely resembles that of aflatoxigenic *A. flavus*. Genome analysis further revealed a nonsense mutation in the *laeA* gene of *A. oryzae* KYI32, a key global regulatory gene of secondary metabolisms in *Aspergillus* [80,81], which is predicted to disrupt normal *laeA* gene expression (Figure 5). Taken together, these findings suggest that the absence of aflatoxin production in *A. oryzae* KYI32, despite its intact ABGC and phylogenetic proximity to *A. flavus*, is likely attributable to regulatory dysfunction rather than structural ABGC gene loss.

Based on the identified different deletions in the *aflT*, *aflU*, and *aflT* genes across species, we designed four primer sets targeting these species-specific variations to distinguish *A. oryzae* from *A. flavus* through differences in PCR amplicon sizes (Table 1 and Figure 3). Previous studies have proposed various PCR-based strategies to distinguish between *A. oryzae* and *A. flavus*, including sequence-level comparison of new genetic markers such as the *cyp51A* gene [48] and real-time detection of expression level of aflatoxin-related genes (*aflS* and *aflR* genes) [82,83]. Alternatively, multiplex PCR assays incorporating four to six primer sets have been employed to discriminate *A. flavus* from other genera or *Aspergillus* sections [84,85,86]. However, these approaches often only discriminate at the genus or section level or require time-consuming procedures such as sequencing or gene expression analysis. To further compare the marker-based discrimination, we additionally evaluated the species-level discrimination of the *cyp51A* gene-targeting primers through in silico primer-genome alignment using the deposited genomes of *A. oryzae* and *A. flavus* [48]. This analysis, however, is predicted to yield limited discriminatory accuracy—approximately 82% for *A. flavus* but only 34% for *A. oryzae*—indicating particularly poor accuracy for *A. oryzae* (Tables S4 and S5). In contrast, the ABGC-targeting primer sets designed in this study enable rapid and reliable species-level identification of *A. oryzae* from *A. flavus*, as well as the strains’ ABGC structure, through a simple PCR amplicon size comparison (Figure 4). PCR assay using ABGC-targeting primer sets for *nuruk*-derived strains confirmed that *A. oryzae* and *A. flavus* could be differentiated with 92% and 100% accuracy, respectively (Table 2 and Table S3). Among the *A. oryzae* strains, 59% showed the AO2 pattern, indicating truncated ABGC, and 32% the AO1 pattern, indicating relatively intact ABGC with species-specific small deletions in the *aflT*, *aflU*, and *aflF* genes. Eight *A. flavus* strains, excluding *A. flavus* KSW16 (AF2 pattern), showed the AF1 pattern characterized by small deletions in the *aflU* and *aflF* genes like *A. oryzae*. This approach enables simultaneous species-level discrimination of *A. oryzae* and *A. flavus* and verification of ABGC structure solely through PCR amplicon size profiling. Importantly, our method bypasses additional sequencing- or expression-based steps and therefore provides a rapid and cost-efficient approach for routine identification.

iPCR analysis enables evaluation of primer performance across expanded genomic datasets by simulating amplification outcomes [87,88]. Although iPCR outputs occasionally differ from experimental PCR due to genome assembly quality or in vivo amplification dynamics, this computational approach provides an efficient strategy for assessing primer specificity and predicting amplification [69,89]. Consistent concordance between large-scale iPCR prediction and experimental PCR results indicates that the designed primer sets exhibit high accuracy and strong applicability for discriminating *A. oryzae* from *A. flavus* (Table 3). Across *A. oryzae* genomes, the predominance of AO-type patterns reflects conserved truncated ABGC structure and species-specific deletions in *aflT* and *aflU*-*aflF* genes. Notably, two *A. oryzae* genomes derived from *nuruk* (KJJ4b and KYI32) showed AF-type patterns, underscoring the evolutionary proximity between *A. oryzae* and *A. flavus*. And this may imply the rare but biologically meaningful presence of regulatory alterations that decouple ABGC integrity from aflatoxin biosynthesis. Furthermore, we compared the iPCR patterns of *A. flavus* genomes with known aflatoxin production status and found that non-aflatoxigenic *A. flavus* may exhibit either AO2 or AF1 patterns (Table 3 and Table S5). This heterogeneity is biologically plausible, as loss of aflatoxin production in *A. flavus* can arise from truncation of the ABGC or from an intact ABGC coupled with defects in regulatory mechanisms, as found in KYI32 [90,91]. Thus, non-aflatoxigenic *A. flavus* may have *A. oryzae*-like or intermediate ABGC profiles, indicating diverse evolutionary pathways leading to aflatoxin non-production. Although this represents a limitation in distinguishing *A. oryzae* from non-aflatoxigenic *A. flavus*, the ABGC-targeted primer sets reliably differentiate *A. oryzae* from aflatoxigenic *A. flavus*, enabling rapid strain verification.

Through sequence-level comparison of the ABGC among *Aspergillus* section *Flavi*, we developed four primer sets targeting species-specific deletions that enable rapid and accurate differentiation between *A. oryzae* and *A. flavus* based on PCR amplicon size. The high accuracy and high concordance between the experimental PCR and iPCR results support the robustness of the primer design and indicate that structural variations in ABGC provide reliable molecular features for species differentiation. Collectively, this ABGC-targeting PCR approach provides an efficient and practical molecular method for distinguishing between two species that share roughly 99.5% genomic similarity but differ critically in food safety relevance.

## 5. Patents

Due to the novelty and applicability of the finding, one patent describing the PCR-based species differentiation of *A. oryzae* and *A. flavus* using four primer sets newly designed in this study is currently filed with the Korean Intellectual Property Office under Application No. 10-2099051 (filed on 2 April 2020) (Title: Composition for detection of strain producing aflatoxin and detection method using the same).

## Figures and Tables

**Table 1:** List of four newly designed primer sets targeting ABGC for the differentiation of *A. oryzae* and *A. flavus* in this study.

Target Region on ABGC	Primer ^2^	Sequence ^3^	GC Content (%)	Ta ^4^ (°C)	Nucleotide Position ^5^ (bp)
*aflYe*	YE1	5′-CATGTGCATCACGGCATGAG-3′	55.0	64.8	304–621
	YE2	5′-ATAACCAGCGGTTCTGCCAA-3′	50.0		
*aflC–aflT*	CT1	5′-TGTGTCGGGGACCTCTATGT-3′	55.0	65.2	70,774–72,500
	CT2	5′-CCCTCCCTGTGTGATGTGTC-3′	60.0		
*aflU–aflF*	UF1	5′-CAACTGCTCGACTGTCGTCT-3′	55.0	64.9	74,498–76,669
	UF2	5′-GAATTGGCTTCGCTCCCTCT-3′	55.0		
AFLA_13945 ^1^	E1	5′-AGGCTCTGGGATCAAAGCTC-3′	55.0	64.2	77,621–77,942
	E2	5′-TAGCGCTGGCATTGAAACAG-3′	50.0		

^1^ The AFLA_13945 (EED51179.1 for *A. oryzae* RIB40) is the last gene in ABGC of *A. flavus* NRRL3357. Since the sequences of AFLA_13945 gene of *A. oryzae* and *A. flavus* are slightly different, two primer sets targeting the AFLA_13945 gene were designed as shown in Table S1. ^2^ Forward primer sequences are written above. ^3^ The primer sequences that exhibit sequence variation between *A. oryzae* and *A. flavus* are highlighted in underline. ^4^ Annealing temperature. ^5^ The start and end positions of the primers within the ABGC were arranged according to *A. flavus* NRRL3357, ranging from *aflYe* to AFLA_13945.

**Table 2:** PCR patterns for *A. oryzae* and *A. flavus* strains isolated from *nuruk*, as determined by PCR analysis using the newly designed primer sets.

PCR Pattern ^1^	No. of Strains ^2^	
	*A. oryzae*	*A. flavus*
AO1	35	0
AO2	64	0
AF1	8	1
AF2	1	8
Total	108	9

^1^ The PCR product size of each pattern is shown in Figure 4. ^2^ Genomic DNA was extracted from 108 strains of *A. oryzae* and 9 strains of *A. flavus* isolated from *nuruk*. PCR analysis was conducted using ABGC-targeting primer sets, and the PCR amplicon size for each strain was confirmed by electrophoresis on a 1% agarose gel. The PCR pattern of each strain was determined according to Figure 4.

**Table 3:** iPCR analysis on *A. oryzae* and *A. flavus* using the newly designed primer sets.

PCR Pattern ^1^	No. of Strains ^2^		
	*A. oryzae*	*A. flavus*	
		Aflatoxigenic	Non-Aflatoxigenic
AO1	43	1	7
AO2	71	6	76
AF1	1	66	57
AF2	1	87	6
Exception	0	1	16
Total	116	161	162

^1^ The PCR product size of each pattern is shown in Figure 4. The exception pattern is those that cannot be classified due to the anomalous sizes of some PCR products analyzed. ^2^ The genome sequences designated as *A. oryzae* (116 genomes) and *A. flavus* (257 genomes) were obtained from the GenBank for iPCR analysis [69]. Additionally, 225 genomes of *A. flavus* were obtained from the AflaPan [52]. iPCR analysis was performed using Fasta v36.3 [65] to determine whether the ABGC-targeting primer sets were aligned to the 116 genomes of *A. oryzae* and 482 genomes of *A. flavus* and to predict the PCR amplicon sizes. The PCR pattern of each strain was determined according to Figure 4. For *A. flavus*, the PCR patterns of 482 genomes tested for aflatoxin production are summarized in Table S5. iPCR analysis showed that among the 116 genomes of *A. oryzae*, only two strains (KJJ4b—AF1, KYI32—AF2) were not classified as AO1 or AO2 pattern.

## Data Availability

The original contributions presented in this study are included in the article/Supplementary Materials. Further inquiries can be directed to the corresponding author.

## Supplementary Materials

The following supporting information can be downloaded at: https://www.mdpi.com/article/10.3390/jof12010010/s1, Figure S1: Gel electrophoresis of PCR products using the four primer sets from 8 reference strains of *Aspergillus* section *Flavi*. PCR analysis was performed on 8 reference strains of *Aspergillus* section *Flavi*: (**a**) *A. oryzae* RIB40, RIB128, (**b**) *A. flavus* ATCC22546, NRRL3357, (**c**) *A. sojae* NBRC4239, NRRL1989, (**d**) *A. parasiticus* ATCC22789, and SU-1. The size of amplified PCR products was confirmed using 1% agarose gel electrophoresis at 100 V for 25 min. The marker (M) for electrophoresis was GeneRuler 1 kb plus DNA ladder. Lane 1, start region amplified using YE1 and YE2 primer set; Lane 2, CT region amplified using CT1 and CT2 primer set; Lane 3, UF region amplified using UF1 and UF2 primer set; and Lane 4, end region amplified using E1 and E2 primer set; Table S1: Sequence variations of ABGC-targeting primser sets designed in this study; Table S2: Comparison of 35 genes of aflatoxin biosynthesis gene clusters of 30 strains of *Aspergillus* section *Flavi*; Table S3: PCR analysis using ABGC-targeting primer sets for 8 reference strains of *Aspergillus* section *Flavi* and 9 strains of *A. flavus* and 108 strains of *A. oryzae* isolated from *nuruk*; Table S4: In silico PCR analysis using ABGC-targeting primer sets for the 116 genomes of *A. oryzae* in the GenBank database, Table S5: In silico PCR analysis using ABGC-targeting primer sets for the 257 genomes of *A. flavus* in the GenBank and 225 from AflaPan. References [48,52,92] are cited in the Supplementary Materials.
