# Supplementary figures and images for: Distinction Between Aspergillus oryzae and Aflatoxigenic Aspergillus flavus by Rapid PCR Method Based on the Comparative Sequence Analysis of the Aflatoxin Biosynthesis Gene Cluster
Source: J Fungi (Basel). 2025 Dec 23;12(1):10. doi: 10.3390/jof12010010 (PMC12842993; doi:10.3390/jof12010010)

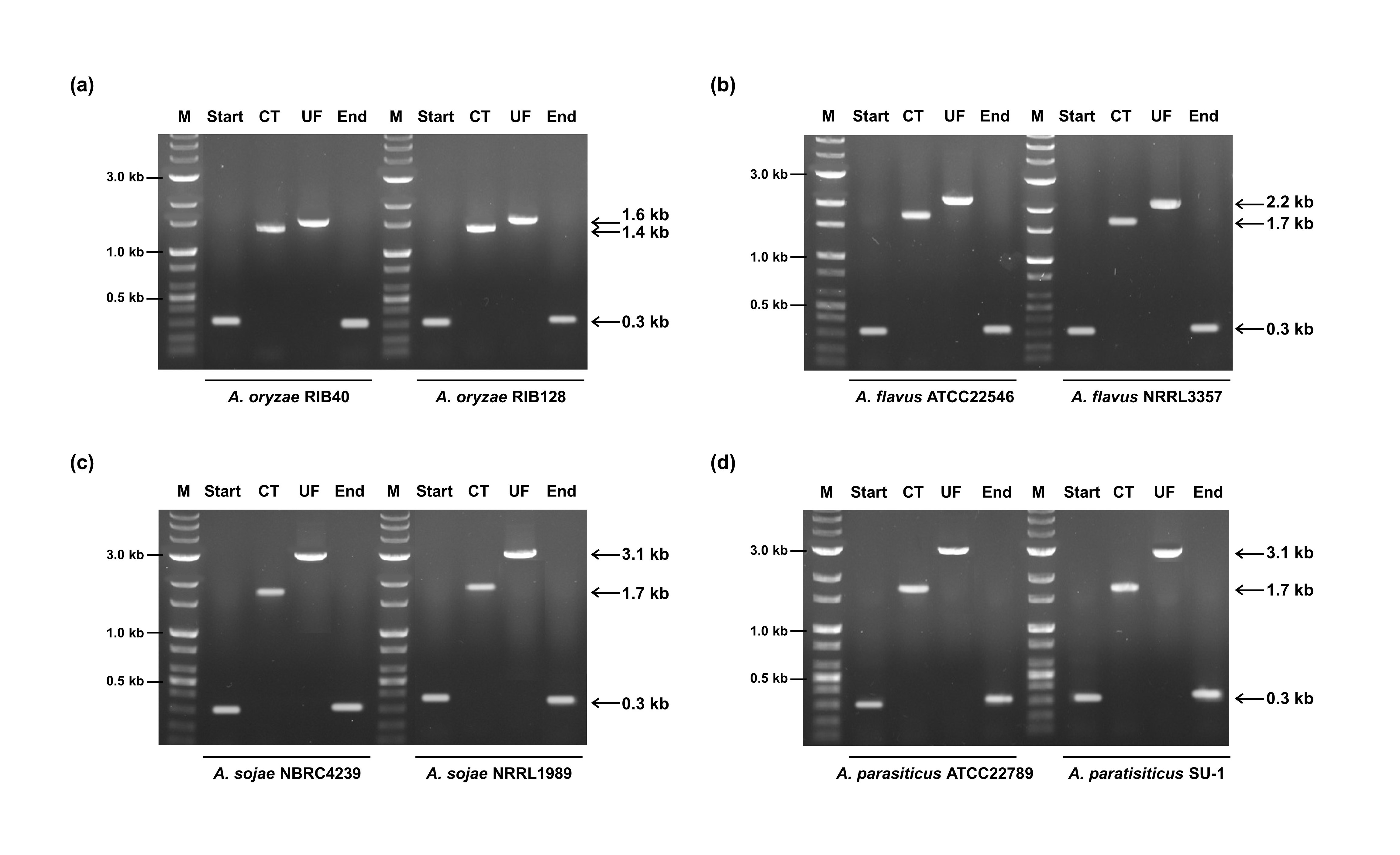

Supplement: Supplementary file 1 [file jof-12-00010-s001.zip › Fig. S1_rev.jpg]
